# Characterization and stability of approximately dual g-frames in Hilbert spaces

**DOI:** 10.1186/s13660-018-1779-7

**Published:** 2018-07-27

**Authors:** Yan-Ling Fu, Wei Zhang

**Affiliations:** 1 Department of Information Engineering, Henan Finance University, Zhengzhou, P.R. China; 2 School of Mathematics and Information Sciences, Henan University of Economics and Law, Zhengzhou, P.R. China

**Keywords:** 42C15, 42C40, Frame, g-frame, Dual g-frame, Approximately dual g-frame

## Abstract

This paper addresses approximately dual g-frames. First, we establish a connection between approximately dual g-frames and dual g-frames and obtain a characterization of approximately dual g-frames. Second, we give results on stability of approximately dual g-frames, which cover the results obtained by other authors.

## Introduction

The notion of frame dates back to Gabor [1] (1946) and Duffin and Schaeffer [2] (1952). Gabor [1] proposed the idea of decomposing a general signal in terms of elementary signals, and Duffin and Schaeffer [2] abstracted “these elementary signals” as the notion of frame. However, the frame theory had not attracted much attention until the celebrated work by Daubechies, Crossman, and Meyer [3] in 1986. So far, the theory of frame has seen great achievements in pure mathematics, science, and engineering ([4–13]). In 2006, Sun [14] introduced a generalized frame (simply g-frame), which covers all other generalizations of frames, for example, fusion frames [15], bounded quasiprojectors [16], and so on. Now, the research of g-frames has obtained many results [17–19]. This paper addresses approximately dual g-frames in Hilbert spaces.

Recall that a sequence $\{f_{i}\}_{i\in I}$ in a separable Hilbert space *H* is a frame if

$$A_{1} \Vert f \Vert ^{2}\leq\sum _{i\in I} \bigl\vert \langle f, f_{n}\rangle \bigr\vert ^{2}\leq B_{1} \Vert f \Vert ^{2} $$

for all $f\in\mathcal{H}$ and some positive constants $A_{1}$, $B_{1}$. Given a frame $\{f_{i}\}_{i\in I}$, another frame $\{h_{i}\}_{i\in I}$ is said to be a dual frame of $\{f_{i}\}_{i\in I}$ if

$$ f=\sum_{i\in I}\langle f, f_{i}\rangle h_{i}, \quad\forall f\in{H}, $$

or, equivalently,

$$ f=\sum_{i\in I}\langle f, h_{i}\rangle f_{i}, \quad\forall f\in{H}. $$

To find the dual frames for a general frame is a fundamental problem in the frame theory. Usually, it is not easy due to involving complicated computation. In 2010, Christensen [20] introduced the notion of approximately dual frames. Bessel sequences $\{f_{i}\}_{i\in I}$ and $\{h_{i}\}_{i\in I}$ in a separable Hilbert space $\mathcal{H}$ are said to be approximately dual frames if

$$\biggl\Vert f-\sum_{i\in I}\langle f, h_{i}\rangle f_{i} \biggr\Vert \leq \Vert f \Vert ,\quad \forall f\in{H}, $$

or

$$\biggl\Vert f-\sum_{i\in I}\langle f, f_{i}\rangle h_{i} \biggr\Vert \leq \Vert f \Vert ,\quad \forall f\in{H}. $$

In 2014, Khosravi et al. [21] first introduced the notion of approximately dual g-frames, which generalize the usual approximately dual frames. They proved that a pair of operator sequences form approximately dual frames if and only if their induced sequences form a pair of approximately dual g-frames. They also obtained some important properties and applications of approximately dual frames. Later, many results on approximately dual g-frames were obtained (see [22, 23]).

Motivated by [21], in this paper, we focus on the characterization and stability of approximately dual g-frames and their connection with dual g-frames. Sect. 2 is an auxiliary one, where we recall some basic notions, properties, and some related results. In Sect. 3, we establish a characterization of approximately dual g-frames and discuss some properties of approximately dual (dual) g-frames. In Sect. 4, we give some stability results of approximately dual g-frames, which cover the results obtained by other authors.

## Preliminaries

We begin with some basic notions and results of g-frames. See [14, 17, 18] for details.

Given separable Hilbert spaces *H* and *V*, let $\{V_{j}:j\in J\}$ be a sequence of closed subspaces of *V* with *J* being a subset of integers $\mathbb {Z}$. The identity operator on *H* is denoted by $I_{H}$. The set of all bounded linear operators from *H* into $V_{j}$ is denoted by $L(H, V_{j})$. Define

$$\bigoplus_{j\in J}V_{j}= \biggl\{ \{a_{j}\}_{j\in J}:a_{j}\in V_{j}, \bigl\Vert \{ a_{j}\}_{j\in J} \bigr\Vert ^{2} =\sum _{j\in J} \Vert a_{j} \Vert ^{2}< \infty \biggr\} . $$

Then $\bigoplus_{j\in J}V_{j}$ is a Hilbert space under the inner product

$$ \bigl\langle \{a_{j}\}_{j\in J}, \{b_{j} \}_{j\in J}\bigr\rangle =\sum_{j\in J}\langle a_{j}, b_{j}\rangle \quad \text{for }\{a_{j} \}_{j\in J}, \{b_{j}\}_{j\in J}\in\bigoplus _{j\in J}V_{j}. $$

Suppose $\{e_{j,k}\}_{k\in K_{j}}$ is an orthonormal basis (simply o.n.b.) for $V_{j}$, where $K_{j}\subset\mathbb{Z}$, $j\in J$. Define $\tilde{e}_{j,k}=\{\delta_{j,i}e_{i,k}\}_{i\in J}$, where *δ* is the Kronecker symbol. Then $\{\tilde{e}_{j,k}\}_{j\in J,k\in K_{j}}$ is an o.n.b. for $\bigoplus_{j\in J}V_{j}$ (see [17]).

### Definition 2.1

([14])

A sequence $\{\Lambda_{j}\in L(H, V_{j})\}_{j\in J}$ is called a g-frame for *H* with respect to (w.r.t.) $\{V_{j}\}_{j\in J}$ if

2.1 $$ A \Vert f \Vert ^{2}\leq\sum _{j\in J} \Vert \Lambda_{j}f \Vert ^{2} \leq B \Vert f \Vert ^{2} $$

for all $f\in H$ and some positive constants $A\leq B$. The numbers *A*, *B* are called the frame bounds. If only the right-hand inequality of (2.1) is satisfied, then $\{\Lambda_{j}\}_{j\in J}$ is called a g-Bessel sequence for *H* w.r.t. $\{V_{j}\}_{j\in J}$ with bound *B*. If $A=B=\lambda$, then $\{\Lambda_{j}\}_{j\in J}$ is called a *λ*-tight g-frame. In addition, if $\lambda=1$, then $\{ \Lambda_{j}\}_{j\in J}$ is called a Parsevel g-frame.

For a g-Bessel sequence $\{\Lambda_{j}\}_{j\in J}$ with bound *B*, the operator

$$T_{\Lambda}: \bigoplus_{j\in J}V_{j} \rightarrow H,\qquad T_{\Lambda}F=\sum_{j\in J} \Lambda_{j}^{\ast}f_{j}, \quad\forall F=\{ f_{j}\}_{j\in J}\in\bigoplus_{j\in J}V_{j}, $$

is well-defined, and its adjoint is given by

$$T_{\Lambda}^{\ast}: H \rightarrow\bigoplus _{j\in J}V_{j},\qquad T_{\Lambda}^{\ast}f=\{ \Lambda_{j}f\}_{j\in J},\quad \forall f\in H. $$

The operator $T_{\Lambda}$ is called the synthesis operator, and $T_{\Lambda}^{\ast}$ is called the analysis operator of $\{\Lambda _{j}\}_{j\in J}$. For g-frame $\{\Lambda_{j}\}_{j\in J}$ with bounds *A* and *B*, the operator

$$S_{\Lambda}: H\rightarrow H,\qquad S_{\Lambda}f=\sum _{j\in J}\Lambda _{j}^{\ast} \Lambda_{j}f, \quad\forall f\in H, $$

is called a g-frame operator of $\{\Lambda_{j}\}_{j\in J}$. It is bounded, invertible, self-adjoint, and positive, and $AI_{H} \leq S_{\Lambda}\leq BI_{H}$. Let $\tilde{\Lambda}_{j}=\Lambda _{j}S_{\Lambda}^{-1}$. Then $\{\tilde{\Lambda}_{j}\}_{j\in J}$ is also a g-frame for *H* w.r.t. $\{V_{j}\}_{j\in J}$ with the g-frame operator $S_{\Lambda}^{-1}$ and frame bounds $\frac{1}{B}$ and $\frac {1}{A}$. $\{\tilde{\Lambda}_{j}\}_{j\in J}$ is called thebcanonical dual g-frame of $\{\Lambda_{j}\}_{j\in J}$ (see [14]).

### Definition 2.2

([14])

Let $\{\Lambda_{j}\}_{j\in J}$ be a g-frame for *H* w.r.t. $\{V_{j}\}_{j\in J}$. A g-frame $\{\Gamma_{j}\}_{j\in J}$ is called an alternate dual g-frame for $\{\Lambda_{j}\}_{j\in J}$ if

$$f=\sum_{j\in J}\Gamma_{j}^{\ast} \Lambda_{j}f,\quad \forall f\in H. $$

Moreover, $\{\Lambda_{j}\}_{j\in J}$ is also an alternate dual g-frame for $\{\Gamma_{j}\}_{j\in J}$, that is,

$$f=\sum_{j\in J}\Lambda_{j}^{\ast} \Gamma_{j}f, \quad\forall f\in H. $$

### Definition 2.3

([20])

Let $\{f_{j}\}_{j\in J}$ and $\{g_{j}\}_{j\in J}$ be two Bessel sequences for *H* with their respective synthesis operators $T_{f}$ and $T_{g}$. We say that$\{f_{j}\}_{j\in J}$ and $\{g_{j}\}_{j\in J}$ are approximately dual frames if $\|I_{H}-T_{f}T^{*}_{g}\|<1$ or $\|I_{H}-T_{g}T^{*}_{f}\|<1$.

It is clear that the operator $T_{f}T^{*}_{g}$ is invertible.

### Definition 2.4

([21])

Let $\{\Lambda_{j}\}_{j\in J}$ and $\{\Gamma_{j}\}_{j\in J}$ be two g-Bessel sequences for *H* w.r.t. $\{V_{j}\}_{j\in J}$ with their respective synthesis operators $T_{\Lambda}$ and $T_{\Gamma}$. Then $\{\Lambda_{j}\}_{j\in J}$ and $\{\Gamma_{j}\} _{j\in J}$ are approximately dual g-frames if $\|I_{H}-T_{\Lambda}T^{*}_{\Gamma}\| <1$ or $\|I_{H}-T_{\Gamma}T^{*}_{\Lambda}\|<1$.

## Dual and approximately dual g-frames

This section focuses on the connection between approximately dual g-frames and dual g-frames and on a characterization of approximately dual g-frames.

### Lemma 3.1

([19])

*Let* $\{\Lambda_{j}\}_{j\in J}$ *and* $\{\Gamma_{j}\}_{j\in J}$ *be two g*-*Bessel sequences for* *H* *w*.*r*.*t*. $\{V_{j}\}_{j\in J}$. *Then the following are equivalent*:

(i) $f=\sum_{j\in J}\Gamma_{j}^{\ast}\Lambda_{j}f$, $\forall f\in H$.
(ii) $f=\sum_{j\in J}\Lambda_{j}^{\ast}\Gamma_{j}f$, $\forall f\in H$.
(iii) $\langle f, g\rangle=\sum_{j\in J}\langle\Lambda_{j}f, \Gamma_{j}g \rangle$, $\forall f,g\in H$.

*In case the equivalent conditions are satisfied*, $\{\Lambda_{j}\} _{j\in J}$ *and* $\{\Gamma_{j}\}_{j\in J}$ *are dual g*-*frames for* *H* *w*.*r*.*t*. $\{V_{j}\}_{j\in J}$.

### Lemma 3.2

([14])

*Let* $\Lambda_{j}\in L(H, V_{j})$ *for every* $j\in J$, *and let* $\{e_{j,k}\}_{k\in K_{j}}$ *be an o*.*n*.*b*. *for* $V_{j}$. *If* $u_{j,k}$ *is defined by* $u_{j,k}=\Lambda_{j}^{\ast}e_{j,k}$, *then* $\{\Lambda_{j}\}_{j\in J}$ *is a g*-*frame* (*g*-*Bessel sequence*) *for* *H* *if and only if* $\{u_{j,k}\}_{j\in J,k\in K_{j}}$ *is a frame* (*Bessel sequence*) *for* *H*.

The following two theorems give a method to construct new dual g-frames (approximately dual g-frames) from given dual g-fromes.

### Theorem 3.1

*Let* $\{\Lambda_{j}\}_{j\in J}$ *and* $\{ \Gamma_{j}\}_{j\in J}$ *be dual g*-*frames for* *H* *w*.*r*.*t*. $\{V_{j}\} _{j\in J}$, *and let* $O_{1}$ *and* $O_{2}$ *be two bounded operators on* *H* *such that* $O_{2}O_{1}^{\ast}=I_{H}$ ($\|I_{H}-O_{2}O_{1}^{\ast}\|<1$). *Then* $\{\Lambda_{j}O_{1}\}_{j\in J}$ *and* $\{\Gamma_{j}O_{2}\}_{j\in J}$ *are dual g*-*frames* (*approximately dual g*-*frames*) *for* *H* *w*.*r*.*t*. $\{ V_{j}\}_{j\in J}$.

### Proof

By a standard argument, $\{\Lambda_{j}\}_{j\in J}$ is a g-Bessel sequence with synthesis operator $T_{\Lambda}$. Since $O_{1}$ is a bounded operator on *H*, we see that $\{\Lambda_{j}O_{1}\}_{j\in J}$ is a g-Bessel sequence with synthesis operator $T_{O\Lambda }=O_{1}T_{\Lambda}$. Similarly, $\{\Gamma_{j}O_{2}\}_{j\in J}$ is also a g-Bessel sequence with synthesis operator $T_{O\Gamma}=O_{2}T_{\Gamma}$. By Lemma 3.1 we have

$$\begin{gathered} T_{O\Gamma}T_{O\Lambda}^{\ast}f=O_{2}T_{\Gamma}T_{\Lambda}^{\ast }O_{1}^{\ast}f=O_{2}O_{1}^{\ast}f=f \\ \bigl( \bigl\Vert I_{H}-T_{O\Gamma}T_{O\Lambda}^{\ast} \bigr\Vert = \bigl\Vert I_{H}-O_{2}T_{\Gamma}T_{\Lambda}^{\ast}O_{1}^{\ast} \bigr\Vert = \bigl\Vert I_{H}-O_{2}O_{1}^{\ast} \bigr\Vert < 1\bigr)\end{gathered} $$

for all $f\in H$. □

### Corollary 3.1

*Let* $\{\Lambda_{j}\}_{j\in J}$ *and* $\{\Gamma_{j}\} _{j\in J}$ *be dual g*-*frames for* *H* *w*.*r*.*t*. $\{V_{j}\}_{j\in J}$, *and let* *T* *be a unitary operator on* *H*. *Then* $\{\Lambda_{j}T\}_{j\in J}$ *and* $\{\Gamma_{j}T\}_{j\in J}$ *are dual g*-*frames* (*approximately dual g*-*frames*) *for* *H* *w*.*r*.*t*. $\{V_{j}\}_{j\in J}$.

### Theorem 3.2

*Assume that* $\{\Lambda_{j}\}_{j\in J}$ *and* $\{\Gamma _{j}\}_{j\in J}$ *are dual g*-*frames for* *H* *w*.*r*.*t*. $\{V_{j}\}_{j\in J}$, *and let* $\{\Lambda_{j}\}_{j\in J}$ *and* $\{\Delta_{j}\}_{j\in J}$ *also be dual g*-*frames for* *H* *w*.*r*.*t*. $\{V_{j}\}_{j\in J}$. *Then for any* $\alpha\in\mathbb{C}$, $\{\Lambda_{j}\}_{j\in J}$ *and* $\{\alpha \Gamma_{j}+(1-\alpha)\Delta_{j}\}_{j\in J}$ *are dual g*-*frames for* *H* *w*.*r*.*t*. $\{V_{j}\}_{j\in J}$.

### Proof

By a standard argument, $\{\alpha\Gamma_{j}+(1-\alpha )\Delta_{j}\}_{j\in J}$ is a g-Bessel sequence for *H* w.r.t. $\{ V_{j}\}_{j\in J}$. By Lemma 3.1 we have

$$\begin{aligned} \sum_{j\in J}\bigl\langle \Lambda_{j}f, \bigl(\alpha\Gamma_{j}+(1-\alpha )\Delta_{j}\bigr)g \bigr\rangle &= \sum_{j\in J}\langle\Lambda_{j}f, \alpha\Gamma_{j}g \rangle +\sum_{j\in J}\bigl\langle \Lambda_{j}f, (1-\alpha)\Delta_{j}g \bigr\rangle \\ &=\bar{\alpha}\sum_{j\in J}\langle \Lambda_{j}f, \Gamma_{j}g \rangle +(1-\bar{\alpha})\sum _{j\in J}\langle\Lambda_{j}f, \Delta_{j}g \rangle \\ &=\bar{\alpha}\langle f, g\rangle+(1-\bar{\alpha})\langle f, g\rangle\\ &= \langle f, g\rangle \end{aligned}$$

for all $f, g\in H$. □

Obviously, if $\{\Lambda_{j}\}_{j\in J}$ and $\{\Gamma_{j}\}_{j\in J}$ are dual g-frames for *H* w.r.t. $\{V_{j}\}_{j\in J}$, then $\{ \Lambda_{j}\}_{j\in J}$ and $\{\Gamma_{j}\}_{j\in J}$ are approximately dual g-frames for *H* w.r.t. $\{V_{j}\}_{j\in J}$. However, the converse is not true in general. The following theorem gives a sufficient condition for approximately dual g-frames to be dual g-frames.

### Theorem 3.3

*Let* $\{\Lambda_{j}\}_{j\in J}$ *and* $\{ \Gamma_{j}\}_{j\in J}$ *be approximately dual g*-*frames for* *H* *w*.*r*.*t*. $\{V_{j}\}_{j\in J}$ *with synthesis operators* $T_{\Lambda}$ *and* $T_{\Gamma}$, *respectively*. *Then* $T_{\Lambda}T_{\Gamma}^{\ast}$ *is invertible*; *furthermore*, *the sequences* $\{\Lambda_{j}\}_{j\in J}$ *and* $\{(T_{\Lambda}T_{\Gamma}^{\ast})^{-1}\Gamma_{j}\}_{j\in J}$ *are dual g*-*frames*.

### Proof

Since $\{\Lambda_{j}\}_{j\in J}$ and $\{\Gamma_{j}\}_{j\in J}$ are approximately dual g-frames for *H* w.r.t. $\{V_{j}\}_{j\in J}$, we have $\|I_{U}-T_{\Lambda}T_{\Gamma}^{\ast}\|<1$, and thus $T_{\Lambda}T_{\Gamma}^{\ast}$ is invertible on *H*. By Lemma 3.1 we have

$$\begin{aligned} \langle f, g\rangle&= \bigl\langle \bigl(T_{\Lambda}T_{\Gamma}^{\ast}\bigr) \bigl(T_{\Lambda}T_{\Gamma}^{\ast}\bigr)^{-1}f, g\bigr\rangle \\ &=\bigl\langle T_{\Gamma}^{\ast}\bigl(T_{\Lambda}T_{\Gamma}^{\ast}\bigr)^{-1}f, T_{\Lambda}^{\ast}g\bigr\rangle \\ &=\sum_{j\in J}\bigl\langle \Gamma_{j}\bigl(T_{\Lambda}T_{\Gamma}^{\ast}\bigr)^{-1}f, \Lambda_{j}g \bigr\rangle \end{aligned} $$

for all $f, g\in H$. □

For Theorem 3.3, a natural question is whether a g-frame always corresponds to an approximately dual g-frame. The following theorem gives an affirmative answer.

### Theorem 3.4

*Let* $\{\Lambda_{j}\}_{j\in J}$ *be a g*-*frame for* *H* *w*.*r*.*t*. $\{V_{j}\}_{j\in J}$ *with the synthesis operator* $T_{\Lambda}$ *and frame bounds* *A* *and* *B*. *Then* $\{B^{-1}\Lambda_{j}\} _{j\in J}$ *is an approximately dual g*-*frame of* $\{\Lambda_{j}\}_{j\in J}$.

### Proof

Note that $\{\Lambda_{j}\}_{j\in J}$ is a g-frame for *H* w.r.t. $\{V_{j}\}_{j\in J}$ and $T_{\Lambda}$ is its synthesis operator. So $\{B^{-1}\Lambda_{j}\}_{j\in J}$ is also g-frame with synthesis operator $B^{-1}T_{\Lambda}$, and

$$\begin{aligned} \bigl\Vert I_{H}-B^{-1}T_{\Lambda}T_{\Lambda}^{\ast}\bigr\Vert &=\sup_{ \Vert f \Vert =1} \bigl\vert \bigl\langle \bigl(I_{H}-B^{-1}T_{\Lambda}T_{\Lambda}^{\ast}\bigr)f, f\bigr\rangle \bigr\vert \\&\leq \frac{B-A}{B}< 1.\end{aligned} $$

It follows that $\{B^{-1}\Lambda_{j}\}_{j\in J}$ is an approximately dual g-frame of $\{\Lambda_{j}\}_{j\in J}$. □

From Theorem 3.4 we know that every g-frame has at least an approximately dual g-frame. Next, we characterize all approximately dual g-frames for a given g-frame. For this purpose, we need to establish some lemmas.

### Lemma 3.3

*Let* $\{\Lambda_{j}\}_{j\in J}$ *be a g*-*frame for* *H* *w*.*r*.*t*. $\{V_{j}\}_{j\in J}$, *let* $T_{\Lambda}$ *be its synthesis operator*, *and let* $\{\tilde{e}_{j,k}\}_{j\in J,k\in K_{j}}$ *be an o*.*n*.*b*. *for* $\bigoplus_{j\in J}V_{j}$. *Then* $\{\Gamma_{j}\}_{j\in J}$ *and* $\{\Lambda_{j}\}_{j\in J}$ *are approximately dual g*-*frames if and only if* $\Gamma_{j}^{\ast}e_{j,k}=T\tilde{e}_{j,k}$ ($\forall j\in J$, $k\in K_{j}$), *where* $T: \bigoplus_{j\in J}V_{j}\rightarrow H$ *is a linear bounded operator such that* $\|I_{H}-TT_{\Lambda}^{\ast}\|<1$.

### Proof

Necessity. Suppose $\{\Gamma_{j}\}_{j\in J}$ is an approximately dual g-frame of $\{\Lambda_{j}\}_{j\in J}$. Then $\{ \Gamma_{j}\}_{j\in J}$ is a g-frame, and $\|I_{H}-T_{\Gamma}T_{\Lambda}^{\ast}\|<1$, where $T_{\Gamma}$ is the synthesis operator of $\{\Gamma _{j}\}_{j\in J}$. Notice that

$$T_{\Gamma}\tilde{e}_{j,k}=T_{\Gamma}\bigl(\{ \delta_{j,i}e_{i,k}\}_{i\in J}\bigr)= \sum _{i\in J}\Gamma_{j}^{\ast}\delta_{j,i}e_{i,k}= \Gamma_{j}^{\ast }e_{j,k}. $$

Denote $T=T_{\Gamma}$. Then $T: \bigoplus_{j\in J}V_{j}\rightarrow H$ is a linear bounded operator satisfying ${\|I_{H}-TT_{\Lambda}^{\ast}\|<1}$ and $\Gamma_{j}^{\ast}e_{j,k}=T\tilde{e}_{j,k}$ for $j\in J$, $k\in K_{j}$.

Next, we prove the converse. Suppose $T: \bigoplus_{j\in J}V_{j}\rightarrow H$ is a linear bounded operator satisfying $\| I_{H}-TT_{\Lambda}^{\ast}\|<1$ and $\Gamma_{j}^{\ast}e_{j,k}=T\tilde {e}_{j,k}$ for $j\in J$, $k\in K_{j}$. Then

$$\begin{aligned} TT_{\Lambda}^{\ast}f& =T \bigl(\{\Lambda_{j}f \}_{j\in J} \bigr) \\ &=T \biggl(\sum_{j\in J}\sum _{k\in K_{j}}\langle\Lambda_{j}f, e_{j,k}\rangle \tilde{e}_{j,k} \biggr) \\ &=\sum_{j\in J}\sum_{k\in K_{j}} \langle\Lambda_{j}f, e_{j,k}\rangle T\tilde{e}_{j,k} \\ &=\sum_{j\in J}\sum_{k\in K_{j}} \langle\Lambda_{j}f, e_{j,k}\rangle \Gamma_{j}^{\ast} e_{j,k} \\ & =\sum_{j\in J}\Gamma_{j}^{\ast}\sum _{k\in K_{j}}\langle\Lambda _{j}f, e_{j,k}\rangle e_{j,k} \\ & =\sum_{j\in J}\Gamma_{j}^{\ast} \Lambda_{j}f\end{aligned} $$

for $f\in H$. Since $\{\tilde{e}_{j,k}\}_{j\in J,k\in K_{j}}$ is an o.n.b. for $\bigoplus_{j\in J}V_{j}$, we have that $\{T\tilde{e}_{j,k}\}_{j\in J,k\in K_{j}}$ is a Bessel sequence for *H*. Let $u_{j,k}=T\tilde {e}_{j,k}$. Then $u_{j,k}=\Gamma_{j}^{\ast}e_{j,k}$. By Lemma 3.2 $\{\Gamma_{j}\}_{j\in J}$ is a g-Bessel sequence for *H* w.r.t. $\{V_{j}\}_{j\in J}$. Let $T_{\Gamma}$ be the synthesis operator of $\{ \Gamma_{j}\}_{j\in J}$. Then $T=T_{\Gamma}$ and $\|I_{H}-T_{\Gamma}T_{\Lambda}^{\ast}\|<1$, and hence $\{\Gamma_{j}\}_{j\in J}$ and $\{ \Lambda_{j}\}_{j\in J}$ are approximately dual g-frames. □

From Lemma 3.3 we know that *T* is very important. The following lemma gives an explicit expression of *T* in Lemma 3.3.

### Lemma 3.4

*Let* $\{\Lambda_{j}\}_{j\in J}$ *be a g*-*frame for* *H* *w*.*r*.*t*. $\{V_{j}\}_{j\in J}$ *with the synthesis operator* $T_{\Lambda}$ *and the frame operator* $S_{\Lambda}$. *Then* $\| I_{H}-TT_{\Lambda}^{\ast}\|<1$ ($T: \bigoplus_{j\in J}V_{j}\rightarrow H$) *if and only if* $T=S_{\Lambda}^{-1}T_{\Lambda}+W(I-T_{\Lambda}^{\ast}QS_{\Lambda}^{-1}T_{\Lambda})$, *where* *I* *is the identity operator on* $\bigoplus_{j\in J}V_{j}$, *and* $W: \bigoplus_{j\in J}V_{j}\rightarrow H$ *and* $Q: H\rightarrow H$ *are linear bounded operators satisfying* $\|WT_{\Lambda}^{\ast}(I_{H}-Q)\|<1$.

### Proof

First, we suppose that $\|I_{H}-TT_{\Lambda}^{\ast}\|<1$ ($T\in L(\bigoplus_{j\in J}V_{j}, H)$). Then $TT_{\Lambda}^{\ast}$ is invertible. Let $W=T$ and $Q=(TT_{\Lambda}^{\ast})^{-1}$. Then

$$\begin{aligned} S_{\Lambda}^{-1}T_{\Lambda}+W\bigl(I-T_{\Lambda}^{\ast}QS_{\Lambda}^{-1}T_{\Lambda}\bigr)&=S_{\Lambda}^{-1}T_{\Lambda}+T \bigl(I-T_{\Lambda}^{\ast}\bigl(TT_{\Lambda}^{\ast}\bigr)^{-1}S_{\Lambda}^{-1}T_{\Lambda}\bigr) \\ &=S_{\Lambda}^{-1}T_{\Lambda}+T-TT_{\Lambda}^{\ast}\bigl(TT_{\Lambda}^{\ast}\bigr)^{-1}S_{\Lambda}^{-1}T_{\Lambda}\\ &= S_{\Lambda}^{-1}T_{\Lambda}+T-S_{\Lambda}^{-1}T_{\Lambda}=T.\end{aligned} $$

Conversely, assume that $T=S_{\Lambda}^{-1}T_{\Lambda}+W(I-T_{\Lambda}^{\ast}QS_{\Lambda}^{-1}T_{\Lambda})$. Then

$$\begin{aligned} TT_{\Lambda}^{\ast}&=\bigl(S_{\Lambda}^{-1}T_{\Lambda}+W \bigl(I-T_{\Lambda}^{\ast}QS_{\Lambda}^{-1}T_{\Lambda}\bigr)\bigr)T_{\Lambda}^{\ast}\\ &=S_{\Lambda}^{-1}T_{\Lambda}T_{\Lambda}^{\ast}+WT_{\Lambda}^{\ast}-WT_{\Lambda}^{\ast}QS_{\Lambda}^{-1}T_{\Lambda}T_{\Lambda}^{\ast}\\ &=I_{U}+WT_{\Lambda}^{\ast}-WT_{\Lambda}^{\ast}Q.\end{aligned} $$

Therefore

$$\bigl\Vert I_{H}-TT_{\Lambda}^{\ast}\bigr\Vert = \bigl\Vert WT_{\Lambda}^{\ast}(I_{H}-Q) \bigr\Vert < 1. $$

 □

Now, we turn to characterizing all approximately dual g-frames for a given g-frame.

### Theorem 3.5

*Let* $\{\Gamma_{j}\in L(H, V_{j})\}$ *be a sequence*, *and let* $\{\Lambda _{j}\}_{j\in J}$ *be a g*-*frame for* *H* *w*.*r*.*t*. $\{V_{j}\}_{j\in J}$ *with the synthesis operator* $T_{\Lambda}$ *and the frame operator* $S_{\Lambda}$. *Then* $\{\Lambda_{j}\}_{j\in J}$ *and* $\{\Gamma_{j}\}_{j\in J}$ *are approximately dual g*-*frames if and only if*

3.1 $$ \Gamma_{j}^{\ast}e_{j,k}=S_{\Lambda}^{-1} \Lambda_{j}^{\ast }e_{j,k}+W\tilde{e}_{j,k}- \sum_{j'\in J}\sum_{k'\in K_{j}}\bigl\langle QS_{\Lambda}^{-1}\Lambda _{j}^{\ast}e_{j,k}, \Lambda_{j'}^{\ast}e_{j',k'}\bigr\rangle W\tilde {e}_{j',k'},\quad \forall j\in J, k\in K_{j}, $$

*where* $W: \bigoplus_{j\in J}V_{j}\rightarrow H$ *and* $Q: H\rightarrow H$ *are linear bounded operators satisfying* $\|WT_{\Lambda}^{\ast}(I_{H}-Q)\|<1$.

### Proof

First, we assume that $\{\Lambda_{j}\}_{j\in J}$ and $\{ \Gamma_{j}\}_{j\in J}$ are approximately dual g-frames. By Lemmas 3.3 and 3.4 we have

3.2 $$ \Gamma_{j}^{\ast}e_{j,k}= \bigl(S_{\Lambda}^{-1}T_{\Lambda}+W\bigl(I-T_{\Lambda}^{\ast}QS_{\Lambda}^{-1}T_{\Lambda}\bigr)\bigr)\tilde {e}_{j,k}, $$

where *I* is the identity operator on $\bigoplus_{j\in J}V_{j}$, and $W: \bigoplus_{j\in J}V_{j}\rightarrow H$ and $Q: H\rightarrow H$ are linear bounded operators satisfying $\|WT_{\Lambda}^{\ast}(I_{U}-Q)\|<1$. Set $z_{j,k}=W\tilde{e}_{j,k}$. We know that $\{z_{j,k}\}_{j\in J, k\in K_{j}}$ is a Bessel sequence for *H*. Using the notations $u_{j,k}:=\Lambda_{j}^{\ast}e_{j,k}$ and $v_{j,k}:=\Gamma_{j}^{\ast }e_{j,k}$, we have

$$\bigl\{ \bigl\langle QS_{\Lambda}^{-1}u_{j,k}, u_{j',k'}\bigr\rangle \bigr\} _{j'\in J,k'\in K_{j}}\in l^{2} $$

for any $j\in J$ and $k\in K_{j}$. So $\sum_{j'\in J}\sum_{k'\in K_{j}}\langle QS_{\Lambda}^{-1}u_{j,k}, u_{j',k'}\rangle z_{j',k'}$ converges unconditionally. By (3.2) we have

$$\begin{aligned} v_{j,k}&=S_{\Lambda}^{-1}T_{\Lambda}\tilde{e}_{j,k}+W\tilde {e}_{j,k}-WT_{\Lambda}^{\ast}QS_{\Lambda}^{-1}T_{\Lambda}\tilde{e}_{j,k} \\ &=S_{\Lambda}^{-1}u_{j,k}+z_{j,k}-WT_{\Lambda}^{\ast}QS_{\Lambda}^{-1}u_{j,k} \\ &=S_{\Lambda}^{-1}u_{j,k}+z_{j,k}-W \biggl(\sum _{j'\in J}\sum_{k'\in K_{j}}\bigl\langle \Lambda_{j'}QS_{\Lambda}^{-1}u_{j,k}, e_{j',k'}\bigr\rangle \tilde{e}_{j',k'} \biggr) \\ &= S_{\Lambda}^{-1}u_{j,k}+z_{j,k}-\sum _{j'\in J}\sum_{k'\in K_{j}}\bigl\langle QS_{\Lambda}^{-1}u_{j,k}, \Lambda_{j'}^{\ast}e_{j',k'} \bigr\rangle W\tilde {e}_{j',k'} \\ &=S_{\Lambda}^{-1}u_{j,k}+z_{j,k}-\sum _{j'\in J}\sum_{k'\in K_{j}}\bigl\langle QS_{\Lambda}^{-1}u_{j,k}, u_{j',k'}\bigr\rangle z_{j',k'}, \end{aligned}$$

that is,

$$\Gamma_{j}^{\ast}e_{j,k}=S_{\Lambda}^{-1} \Lambda_{j}^{\ast }e_{j,k}+W\tilde{e}_{j,k}- \sum_{j'\in J}\sum_{k'\in K_{j}}\bigl\langle QS_{\Lambda}^{-1}\Lambda _{j}^{\ast}e_{j,k}, \Lambda_{j'}^{\ast}e_{j',k'}\bigr\rangle W \tilde{e}_{j',k'} $$

for all $j\in J$, $k\in K_{j}$.

Now we prove the converse. Assume that (3.1) holds. For any $f\in H$, using the notations $u_{j,k}:=\Lambda_{j}^{\ast}e_{j,k}$, $v_{j,k}:=\Gamma_{j}^{\ast}e_{j,k}$, and $z_{j,k}:=W\tilde{e}_{j,k}$, by a standard argument we get that $\sum_{j\in J}\sum_{k\in K_{j}}\langle f, u_{j,k}\rangle S^{-1}_{\Lambda}u_{j,k}$ converges unconditionally to *f*. Therefore

$$\begin{aligned} \sum_{j\in\mathcal{J}}\Gamma_{j}^{\ast} \Lambda_{j}f&= \sum_{j\in\mathcal{J}} \Gamma_{j}^{\ast} \sum_{k\in\mathcal{K}_{j}}\langle \Lambda_{j}f, e_{j,k}\rangle e_{j,k} \\ &=\sum_{j\in\mathcal{J}} \sum_{k\in\mathcal{K}_{j}} \bigl\langle f, \Lambda_{j}^{\ast }e_{j,k}\bigr\rangle \Gamma_{j}^{\ast}e_{j,k} \\ &= \sum_{j\in\mathcal{J}} \sum_{k\in\mathcal{K}_{j}} \langle f, u_{j,k}\rangle v_{j,k} \\ &=\sum_{j\in\mathcal{J}} \sum_{k\in\mathcal{K}_{j}} \langle f, u_{j,k}\rangle \biggl(S^{-1}_{\Lambda}u_{j,k}+z_{j,k}- \sum_{j^{\prime}\in\mathcal{J}} \sum_{k^{\prime}\in\mathcal{K}_{j}}\bigl\langle QS^{-1}_{\Lambda }u_{j,k}, u_{j^{\prime},k^{\prime}}\bigr\rangle z_{j^{\prime},k^{\prime }} \biggr) \\ &= \sum_{j\in\mathcal{J}} \sum_{k\in\mathcal{K}_{j}} \langle f, u_{j,k}\rangle S^{-1}_{\Lambda }u_{j,k}+ \sum_{j\in\mathcal{J}} \sum_{k\in\mathcal{K}_{j}} \langle f, u_{j,k}\rangle z_{j,k} \\ &\quad{} - \sum_{j\in\mathcal{J}} \sum_{k\in\mathcal{K}_{j}} \langle f, u_{j,k}\rangle\sum_{j^{\prime }\in\mathcal{J}} \sum _{k^{\prime}\in\mathcal{K}_{j}}\bigl\langle QS^{-1}_{\Lambda }u_{j,k}, u_{j^{\prime},k^{\prime}}\bigr\rangle z_{j^{\prime},k^{\prime }} \\ &= f+\sum_{j\in\mathcal{J}} \sum_{k\in\mathcal{K}_{j}} \langle f, u_{j,k}\rangle z_{j,k}-\sum _{j\in\mathcal{J}} \sum_{k\in\mathcal{K}_{j}} \biggl\langle Q \sum_{j^{\prime}\in \mathcal{J}} \sum_{k^{\prime}\in\mathcal{K}_{j}} \langle f, u_{j,k}\rangle S^{-1}_{\Lambda}u_{j,k}, u_{j^{\prime},k^{\prime}} \biggr\rangle z_{j^{\prime},k^{\prime}} \\ &=f+\sum_{j\in\mathcal{J}} \sum_{k\in\mathcal{K}_{j}} \langle f, u_{j,k}\rangle z_{j,k}- \sum _{j\in\mathcal{J}} \sum_{k\in\mathcal{K}_{j}}\langle Qf, u_{j^{\prime},k^{\prime }}\rangle z_{j^{\prime},k^{\prime}} \\ &= f+\sum_{j\in\mathcal{J}} \sum_{k\in\mathcal{K}_{j}} \langle f-Qf, u_{j,k}\rangle z_{j,k}\end{aligned} $$

for all $f\in H$. Next, we prove that $\{\Gamma_{j}\}_{j\in J}$ is a g-Bessel sequence for *H* w.r.t. $\{V_{j}\}_{j\in J}$. Indeed,

$$\begin{aligned} \sum_{j\in J} \Vert \Gamma_{j}f \Vert ^{2}&=\sum_{j\in\mathcal{J}} \sum _{k\in K_{j}} \bigl\vert \langle\Gamma_{j}f, e_{j,k}\rangle \bigr\vert ^{2} \\ &=\sum_{j\in J} \sum_{k\in K_{j}} \bigl\vert \langle f, v_{j,k}\rangle \bigr\vert ^{2} \\ &= \sum_{j\in J} \sum_{k\in K_{j}} \biggl\vert \biggl\langle f, S^{-1}_{\Lambda }u_{j,k}+z_{j,k}- \sum_{j^{\prime}\in J} \sum_{k^{\prime}\in K_{j}}\bigl\langle QS^{-1}_{\Lambda}u_{j,k}, u_{j^{\prime},k^{\prime}}\bigr\rangle z_{j^{\prime},k^{\prime}} \biggr\rangle \biggr\vert ^{2} \\ &\leq C_{1} \biggl(\sum_{j\in\mathcal{J}} \sum _{k\in\mathcal{K}_{j}} \bigl\vert \bigl\langle f, S^{-1}_{\Lambda }u_{j,k}\bigr\rangle \bigr\vert ^{2}+\sum_{j\in J} \sum _{k\in K_{j}} \bigl\vert \langle f, z_{j,k}\rangle \bigr\vert ^{2} \\ &\quad{} +\sum_{j\in J} \sum_{k\in K_{j}} \biggl\vert \biggl\langle Q^{\ast}\sum_{j^{\prime}\in J} \sum_{k^{\prime}\in K_{j}}\langle f, z_{j^{\prime},k^{\prime }}\rangle u_{j^{\prime},k^{\prime}},S^{-1}_{\Lambda}u_{j,k}\biggr\rangle \biggr\vert ^{2} \biggr) \\ &\leq C_{2} \biggl( \Vert f \Vert ^{2}+ \biggl\Vert Q^{\ast}\sum_{j^{\prime}\in J} \sum _{k^{\prime}\in K_{j}}\langle f, z_{j^{\prime},k^{\prime }}\rangle u_{j^{\prime},k^{\prime}} \biggr\Vert ^{2} \biggr) \\ &\leq C_{3} \biggl( \Vert f \Vert ^{2}+\sum _{j^{\prime}\in J} \sum_{k^{\prime}\in K_{j}} \bigl\vert \langle f, z_{j^{\prime}k^{\prime }}\rangle \bigr\vert ^{2} \biggr) \\ &\leq C_{4} \Vert f \Vert ^{2} \end{aligned}$$

for all $f\in H$, where $C_{1}$, $C_{2}$, $C_{3}$, and $C_{4}$ are different positive constants. Let $T_{\Gamma}$ be the synthesis operator of $\{\Gamma_{j}\}_{j\in J}$. Then

$$\begin{aligned} \bigl\Vert \bigl(I_{H}-T_{\Gamma}T^{\ast}_{\Lambda} \bigr)f \bigr\Vert &= \biggl\Vert \sum_{j\in J} \sum _{k\in K_{j}}\langle f-Qf, u_{j,k}\rangle z_{j,k} \biggr\Vert \\ &= \biggl\Vert \sum_{j\in J} \sum _{k\in K_{j}}\langle f-Qf, u_{j,k}\rangle W \tilde{e}_{j,k} \biggr\Vert \\ &= \biggl\Vert W\sum_{j\in J} \sum _{k\in K_{j}}\langle f-Qf, u_{j,k}\rangle \tilde{e}_{j,k} \biggr\Vert \\ &= \biggl\Vert W\sum_{j\in J} \sum _{k\in K_{j}}\bigl\langle \Lambda_{j}(f-Qf),e_{j,k} \bigr\rangle \tilde {e}_{j,k} \biggr\Vert \\ &= \bigl\Vert WT^{\ast}_{\Lambda}(f-Qf) \bigr\Vert \\ &\leq \bigl\Vert WT^{\ast}_{\Lambda}(I_{H}-Q) \bigr\Vert \Vert f \Vert \end{aligned}$$

for all $f\in H$. Therefore $\|I_{H}-T_{\Gamma}T^{\ast}_{\Lambda}\|<1$, and thus $\{ \Lambda_{j}\}_{j\in J}$ and $\{\Gamma_{j}\}_{j\in J}$ are approximately dual g-frames. □

## Perturbations of approximately dual g-frames

The stability of frames is of great importance in frame theory, and it is studied widely by a lot of authors ([4, 18]). In this section, we show that, under some conditions, approximately dual g-frames and g-frames are stable under some perturbations. We first introduce some lemmas.

### Lemma 4.1

([17])

*Let* $\{\Lambda_{j}\}_{j\in J}$ *be a g*-*frame for* *H* *w*.*r*.*t*. $\{V_{j}\} _{j\in J}$ *with bounds* *A* *and* *B*, $\lambda_{1}, \lambda_{2}\in(-1, 1)$, $\mu\geq0$, *and* $\max\{\lambda_{1} + \frac{\mu}{\sqrt {A}},\lambda_{2}\}< 1$. *If* $\{\Gamma_{j}\in L(H, V_{j})\}_{j\in J}$ *satisfies*

$$\biggl\Vert \sum_{j\in J_{1}}(\Lambda_{j}- \Gamma_{j})^{\ast}g_{j} \biggr\Vert \leq \lambda_{1} \biggl\Vert \sum_{j\in J_{1}} \Lambda_{j}^{\ast}g_{j} \biggr\Vert + \lambda_{2} \biggl\Vert \sum_{j\in J_{1}} \Gamma_{j}^{\ast}g_{j} \biggr\Vert +\mu \biggl(\sum _{j\in J_{1}} \Vert g_{j} \Vert ^{2} \biggr)^{\frac{1}{2}} $$

*for an arbitrary finite subset* $J_{1}\subset J$ *and* $g_{j}\in V_{j}$, *then* $\{ \Gamma_{j}\}_{j\in J}$ *is a g*-*frame for* *H* *w*.*r*.*t*. $\{V_{j}\}_{j\in J}$ *with bounds*

$$\frac{((1-\lambda_{1})\sqrt{A}-\mu)^{2}}{(1+\lambda_{2})^{2}},\qquad \frac{((1+\lambda_{1})\sqrt{B}+\mu)^{2}}{(1-\lambda_{2})^{2}}. $$

### Lemma 4.2

([14])

*Let* $\{\Lambda_{j}\}_{j\in J}$ *be a g*-*frame for* *H* *w*.*r*.*t*. $\{V_{j}\} _{j\in J}$. *Then for* $g_{j}\in V_{j}$ *satisfying* $f=\sum_{j\in J}\Lambda _{j}^{\ast}g_{j}$, *we have*

$$\sum_{j\in J} \Vert g_{j} \Vert ^{2}\geq\sum_{j\in J} \Vert \tilde{ \Lambda}_{j}f \Vert ^{2}. $$

### Lemma 4.3

([14])

$\{\Lambda_{j}\}_{j\in J}$ *is a g*-*Bessel sequence with an upper bound* *B* *if and only if*

$$\biggl\Vert \sum_{j\in{J}_{1}}\Lambda_{j}^{\ast}g_{j} \biggr\Vert ^{2}\leq B\sum_{j\in{J}_{1}} \Vert g_{j} \Vert ^{2},\quad g_{j}\in V_{j}, $$

*where* $J_{1}$ *is an arbitrary finite subset of* *J*.

### Theorem 4.1

*Let* $\Lambda_{j}\in L(H, V_{j})$, *let* $\{\Gamma_{j}\}_{j\in J}$ *be a g*-*frame for* *H* *w*.*r*.*t*. $\{V_{j}\}_{j\in J}$ *with bounds* *A* *and* *B* *and the synthesis operator* $T_{\Lambda}$, *and let* $\{\Delta_{j}\}_{j\in J}$ *be alternate dual for* $\{\Gamma_{j}\}_{j\in J}$ *with the upper bound* *C* *and the synthesis operator* $T_{\Delta}$. *Assume that there are constants* $\lambda_{1}$, $\mu\geq0$, *and* $0\leq\lambda_{2}<1$ *satisfying*

4.1 $$ \biggl\Vert \sum_{j\in J_{1}}( \Gamma_{j}-\Lambda _{j})^{\ast}g_{j} \biggr\Vert \leq\lambda_{1} \biggl\Vert \sum _{j\in J_{1}}\Gamma _{j}^{\ast}g_{j} \biggr\Vert +\lambda_{2} \biggl\Vert \sum _{j\in J_{1}}\Lambda _{j}^{\ast}g_{j} \biggr\Vert +\mu \biggl(\sum_{j\in J_{1}} \Vert g_{j} \Vert ^{2} \biggr)^{\frac{1}{2}}, $$

*where* $J_{1}$ *is an arbitrary finite subset of* *J*, *and* $g_{j}\in V_{j}$. *If*

$$\lambda_{1}+\lambda_{2}\sqrt{BC} \biggl(1+\frac{\lambda_{1}+ \lambda_{2}+\frac{\mu}{\sqrt{B}}}{1-\lambda_{2}} \biggr)+\mu\sqrt{C}< 1, $$

*then* $\{\Lambda_{j}\}_{j\in J}$ *and* $\{\Delta_{j}\}_{j\in J}$ *are approximately dual g*-*frames*.

### Proof

By Lemma 4.2 we have $C\geq\frac{1}{A}$ and $BC\geq\frac{B}{A}\geq1$. Note that

$$\lambda_{1}+\lambda_{2}\sqrt{BC} \biggl(1+\frac{\lambda_{1}+ \lambda_{2}+\frac{\mu}{\sqrt{B}}}{1-\lambda_{2}} \biggr)+\mu\sqrt{C}< 1. $$

It follows that $\lambda_{1}+\frac{\mu}{\sqrt{A}}<1$. By Lemma 4.1 $\{\Lambda_{j}\}_{j\in J}$ is a g-frame for *H* w.r.t. $\{ V_{j}\}_{j\in J}$ with bounds

$$A\biggl(1-\frac{\lambda_{1}+\lambda_{2}+\frac{\mu}{\sqrt{A}}}{1+\lambda _{2}}\biggr)^{2},\qquad B\biggl(1+\frac{\lambda_{1}+\lambda_{2}+\frac{\mu}{\sqrt {B}}}{1-\lambda_{2}} \biggr)^{2}. $$

Denote by $T_{\Lambda}$ the synthesis operator of $\{\Lambda_{j}\} _{j\in J}$. From (4.1) we have

4.2 $$ \Vert T_{\Gamma}c-T_{\Lambda}c \Vert \leq \lambda_{1} \Vert T_{\Gamma}c \Vert +\lambda_{2} \Vert T_{\Lambda}c \Vert +\mu \Vert c \Vert _{\bigoplus_{j\in J}V_{j}} $$

for any $c=\{c_{j}\}_{j\in J}\in\bigoplus_{j\in J}V_{j}$. Take $c=T_{\Delta}^{\ast}f$ in (4.2). Then

$$\begin{aligned} \bigl\Vert \bigl(I_{H}-T_{\Lambda}T_{\Delta}^{\ast} \bigr)f \bigr\Vert &\leq \lambda_{1} \Vert f \Vert + \lambda_{2} \bigl\Vert T_{\Lambda}T_{\Delta}^{\ast}f \bigr\Vert +\mu \bigl\Vert T_{\Delta}^{\ast}f \bigr\Vert _{\bigoplus_{j\in J}V_{j}} \\ &\leq\lambda _{1} \Vert f \Vert +\lambda_{2}\sqrt{C} \Vert T_{\Lambda} \Vert \Vert f \Vert +\mu\sqrt{C} \Vert f \Vert \\ &\leq\lambda_{1} \Vert f \Vert +\lambda_{2}\sqrt{BC} \biggl(1+\frac{\lambda_{1}+\lambda_{2}+\frac{\mu}{\sqrt {B}}}{1-\lambda_{2}} \biggr) \Vert f \Vert +\mu\sqrt{C} \Vert f \Vert \\ &= \biggl(\lambda_{1}+\lambda_{2}\sqrt{BC} \biggl(1+ \frac{\lambda_{1}+\lambda_{2}+\frac{\mu}{\sqrt {B}}}{1-\lambda_{2}} \biggr) +\mu\sqrt{C} \biggr) \Vert f \Vert \end{aligned}$$

for any $f\in H$. So

$$\bigl\Vert I_{H}-T_{\Lambda}T_{\Delta}^{\ast} \bigr\Vert \leq\lambda_{1}+\lambda _{2}\sqrt{BC} \biggl(1+ \frac{\lambda_{1}+\lambda_{2}+\frac{\mu}{\sqrt {B}}}{1-\lambda_{2}} \biggr) +\mu\sqrt{C}< 1. $$

Thus $\{\Lambda_{j}\}_{j\in J}$ and $\{\Delta_{j}\}_{j\in J}$ are approximately dual g-frames if $\lambda_{1}+\lambda_{2}\sqrt{BC} (1+\frac{\lambda_{1}+ \lambda_{2}+\frac{\mu}{\sqrt{B}}}{1-\lambda_{2}} )+\mu\sqrt{C}<1$. □

From Theorem 4.1 we can obtain immediately the following corollary.

### Corollary 4.1

*Let* $\Lambda_{j}\in L(H, V_{j})$, *let* $\{\Gamma_{j}\}_{j\in J}$ *be a g*-*frame for* *H* *w*.*r*.*t*. $\{V_{j}\}_{j\in J}$ *with bounds* *A* *and* *B* *and the synthesis operator* $T_{\Gamma}$, *and let* $\{\Delta_{j}\}_{j\in J}$ *be the canonical dual for* $\{\Gamma_{j}\}_{j\in J}$ *with the synthesis operator* $T_{\Delta}$. *Suppose that there are constants* $\lambda_{1}$, $\mu\geq0$, *and* $0\leq\lambda_{2}<1$ *such that*

4.3 $$ \biggl\Vert \sum_{j\in J_{1}}(\Gamma_{j}- \Lambda_{j})^{\ast }g_{j} \biggr\Vert \leq \lambda_{1} \biggl\Vert \sum_{j\in J_{1}} \Gamma_{j}^{\ast }g_{j} \biggr\Vert + \lambda_{2} \biggl\Vert \sum_{j\in J_{1}} \Lambda_{j}^{\ast }g_{j} \biggr\Vert +\mu \biggl( \sum_{j\in J_{1}} \Vert g_{j} \Vert ^{2} \biggr)^{\frac{1}{2}}, $$

*where* $J_{1}$ *is an arbitrary finite subset of* *J*, *and* $g_{j}\in V_{j}$. *If* $\lambda_{1}+\lambda_{2}\sqrt{\frac{B}{A}} (1+\frac{\lambda_{1}+ \lambda_{2}+\frac{\mu}{\sqrt{B}}}{1-\lambda_{2}} )+\frac{\mu }{\sqrt{A}}<1$, *then* $\{\Lambda_{j}\}_{j\in J}$ *and* $\{\Delta_{j}\} _{j\in J}$ *are approximately dual g*-*frames*.

Note that $\{\Gamma_{j}\}_{j\in J}$ is a Parseval g-frame for *H* w.r.t. $\{V_{j}\}_{j\in J}$. Then $\{\Gamma_{j}\}_{j\in J}$ is the canonical dual for itself. We have the following:

### Corollary 4.2

*Let* $\Lambda_{j}\in L(H, V_{j})$, *and let* $\{\Gamma_{j}\}_{j\in J}$ *be a Parseval g*-*frame for* *H* *w*.*r*.*t*. $\{V_{j}\}_{j\in J}$. *Assume that there are constants* $\lambda,\mu\geq0$ *such that*

4.4 $$ \biggl\Vert \sum_{j\in J_{1}}(\Gamma_{j}- \Lambda_{j})^{\ast }g_{j} \biggr\Vert \leq\lambda \biggl\Vert \sum_{j\in J_{1}}\Gamma_{j}^{\ast }g_{j} \biggr\Vert +\mu \biggl(\sum_{j\in J_{1}} \Vert g_{j} \Vert ^{2} \biggr)^{\frac{1}{2}} $$

*for an arbitrary finite subset* $J_{1}\subset J$ *and* $g_{j}\in V_{j}$. *If* $\lambda+\mu<1$, *then* $\{\Lambda_{j}\}_{j\in J}$ *and* $\{\Gamma_{j}\} _{j\in J}$ *are approximately dual g*-*frames*.

### Corollary 4.3

*Let* $\{\Lambda_{j}\in L(H, V_{j})\}_{j\in J}$ *be a sequence*, *and let* $\{ \Gamma_{j}\}_{j\in J}$ *be a g*-*frame for* *H* *w*.*r*.*t*. $\{V_{j}\}_{j\in J}$. *Also*, *let* $\{\Delta_{j}\}_{j\in J}$ *be an alternate dual for* $\{ \Gamma_{j}\}_{j\in J}$ *with the upper bound* *C*. *If there exists a constant* *R* *such that* $CR<1$ *and*

4.5 $$ \sum_{j\in J} \bigl\Vert ( \Gamma_{j}-\Lambda_{j})f \bigr\Vert ^{2}\leq R \Vert f \Vert ^{2},\quad \forall f\in H, $$

*then* $\{\Lambda_{j}\}_{j\in J}$ *and* $\{\Delta_{j}\}_{j\in J}$ *are approximately dual g*-*frames*.

### Proof

Take $\lambda_{1}=\lambda_{2}=0$ and $\mu=\sqrt{R}$ in Theorem 4.1. From Lemma 4.3 we know that (4.1) is equivalent to (4.5). Since $CR<1$, we have that $\{\Lambda_{j}\}_{j\in J}$ and $\{\Delta _{j}\}_{j\in J}$ are approximately dual g-frames. □

### Remark 4.1

Corollary 4.1 and Corollary 4.3 are Proposition 3.10(i) and Theorem 3.1(i) in [21], respectively. They are particular cases of our Theorem 4.1.

### Theorem 4.2

*Let* $\{\Lambda_{j}\}_{j\in J}$ *be a g*-*frame for* *H* *w*.*r*.*t*. $\{V_{j}\}_{j\in J}$ *with synthesis operator* $T_{\Lambda}$ *and bounds* *A* *and* *B*. *Assume that* $\Gamma_{j}\in L(H, V_{j})$ *for all* $j\in J$ *and there exist constants* $\lambda_{1}, \lambda _{2}, \mu\geq0$ *such that*

4.6 $$ \biggl\Vert \sum_{j\in J_{1}}(\Lambda_{j}- \Gamma_{j})^{\ast }g_{j} \biggr\Vert \leq \lambda_{1} \biggl\Vert \sum_{j\in J_{1}} \Lambda_{j}^{\ast }g_{j} \biggr\Vert + \lambda_{2} \biggl\Vert \sum_{j\in J_{1}} \Gamma_{j}^{\ast }g_{j} \biggr\Vert +\mu \biggl(\sum _{j\in J_{1}} \Vert g_{j} \Vert ^{2} \biggr)^{\frac{1}{2}} $$

*for an arbitrary finite subset* $J_{1}\subset J$ *and* $g_{j}\in V_{j}$. *If* $\lambda_{1}+\frac{\mu}{\sqrt{A}}<1$ *and* $\lambda_{2}+(\lambda_{1}\sqrt {\frac{B}{A}}+\frac{\mu}{\sqrt{A}}) \frac{1+\lambda_{2}}{ 1-(\lambda_{1}+\frac{\mu}{\sqrt{A}})}<1$, *then* $\{\Gamma_{j}\} _{j\in J}$ *is a g*-*frame for* *H* *w*.*r*.*t*. $\{V_{j}\}_{j\in J}$, *and* $\{ \tilde{\Gamma}_{j}\}_{j\in J}$ *and* $\{\Lambda_{j}\}_{j\in J}$ *are approximately dual g*-*frames*.

### Proof

We can prove the theorem by an argument similar to that of Theorem 4.1. □

## Conclusions

For a given frame, it is usually not easy to find a dual frame. The notion of approximately dual frames was introduced by Christensen in 2010. It is a generalization of dual frames. In this paper, on one hand, we obtain the link between approximately dual g-frames and dual g-frames and characterize approximately dual g-frames. On the other hand, we give stability results of approximately dual g-frames, which cover the results obtained by other authors.
